# Bringing therapy home: Exploring parents’ experiences of telehealth for children with developmental coordination disorder

**DOI:** 10.1177/03080226231181018

**Published:** 2023-07-14

**Authors:** Aisling Bourke, Una O’Connor, Bryan Boyle, Jessica Kennedy, Helen Lynch

**Affiliations:** Department of Occupational Therapy, College of Medicine and Health, University College Cork, Cork, Ireland

**Keywords:** Telehealth, developmental coordination disorder, occupational therapy

## Abstract

**Introduction:**

Developmental coordination disorder (DCD) significantly impacts a child’s motor skills and ability to learn and perform self-care and academic tasks. Telehealth is a rapidly emerging service delivery model, ensuring expanded access to services and continuity of care. Many benefits to the use of telehealth have been identified; yet, there is a dearth of published evidence available on the experiences of parents of children with DCD.

**Aim:**

The aim of this study was to explore parents’ experiences of implementing a telehealth motor skills programme for their child with DCD.

**Method:**

This study utilised a qualitative descriptive approach to best capture parents’ experiences of a telehealth programme. Data were collected through in-depth, semi-structured interviews with eight participants and analysed using thematic analysis. Measures to ensure the trustworthiness of the study were observed within the naturalistic paradigm comprising criteria of credibility, transferability, dependability and confirmability.

**Findings:**

This study uncovered three major themes: (1) Parents in the dark, (2) telehealth in the family and (3) telehealth: what success looks like. Ten further subthemes highlighted the factors for success and parents’ considerations for future service delivery.

**Conclusion:**

Challenges for parents in accessing traditional occupational therapy services present an opportunity to explore alternative means of delivery such as telehealth. Parents, however, are clear in their preference for a blended approach for future services.

## Introduction

Developmental coordination disorder (DCD) is a neurodevelopmental disability that affects approximately 5–6% of school-aged children globally (American Psychiatric Association (APA), 2013). DCD significantly impacts a child’s motor skills and ability to learn and perform self-care and academic tasks (Zwicker et al., 2018). If these needs go unmet, children with DCD are at risk of developing profound secondary physical and psychosocial issues that can follow them into adulthood (Tal-Saban et al., 2014). These may include social isolation (Missiuna et al., 2015), anxiety and depression (Omer et al., 2019), reduced physical fitness (Zwicker et al., 2018) and poorer educational outcomes (Harrowell et al., 2018). Conversely, parenting a child with motor difficulties has been linked to increased stress, lower sense of well-being and financial difficulties (Soriano et al., 2015). Parental concerns of DCD are often disregarded by health professionals with many clinicians believing that the child will inevitably ‘grow out of it’ (Missiuna et al., 2006). Despite the prevalence of DCD (APA 2013), this population group often face long waiting times and receive insufficient in-person services (Missiuna et al., 2015). This is not surprising given that children who experience mild motor difficulties are often underserved, underdiagnosed (Blank et al., 2019) and underrepresented in the literature (Camden et al., 2015).

Typical settings of occupational therapy intervention for children with DCD include the clinic primarily, along with the home, and school (Camden et al., 2015). Informed by best evidence, therapy interventions typically seek to address the needs of the child, through various intervention approaches that are grouped into three levels: (1) body function and structure oriented, (2) activity oriented and (3) participation oriented (Blank et al., 2019). However, the recent coronavirus disease 2019 pandemic saw clinicians adopt telehealth as an alternative solution for delivering such interventions safely at a distance (Hoel et al., 2021). Telehealth is defined as ‘the application of evaluative, consultative, preventative, and therapeutic services delivered through telecommunication and information technologies’ (American Occupational Therapy Association (AOTA), 2018:1). Globally, telehealth has been well supported in a wide variety of occupational therapy settings (AOTA, 2018; Zylstra, 2013). Yet, it removes the possibility for interventions where the therapist works directly with the child to work on tasks or on motor training which are common approaches in addressing DCD (Blank et al., 2019). Instead, therapy is focused on indirect approaches which rely significantly on carer engagement. As healthcare professionals, occupational therapists have a duty to embrace telehealth, capitalise on its unique benefits, and understand its limitations (Rosenbaum et al., 2021). To successfully implement telehealth as an effective component of paediatric occupational therapy practice (Zylstra, 2013), understanding parents’ needs and learning styles is paramount. The purpose of this study is to explore the experiences of parents who have accessed and engaged with a telehealth solution developed to support families of children with DCD.

## Literature review

Telehealth offers occupational therapists the opportunity to provide services remotely to clients, allowing intervention to take place in the client’s natural context (AOTA, 2018). Telehealth takes the form of two main methods of service delivery: synchronous (live) and asynchronous (store and forward) technologies with video conferencing being the most cited in the literature (Cason, 2014). An evidence-based review of telehealth in paediatric occupational therapy suggests that the impact for parents is significant in terms of capacity building and strengthening their resources (Zylstra, 2013). Two qualitative studies examining parental perspectives of telehealth revealed that telehealth interventions were preferable to traditional therapy approaches, as they integrated well into family life (Eguia and Capio, 2021; Wallisch et al., 2019). Telehealth allowed families to incorporate new knowledge in the home environment by embedding strategies into existing family routines to support their child (Johnston, 2019). This appeared to enhance parents’ problem-solving abilities in devising new strategies and situations with their child (Wallisch et al., 2019). Parental engagement in telehealth has also been linked to feelings of empowerment and an increased understanding of both their child’s behaviours and strengths (Eguia and Capio, 2021).

Studies conclude that parents’ experience and clinical outcomes of telehealth differed from receiving in-person therapy for their child (Wallisch et al., 2019). While challenges exist with poor internet connection (Eguia and Capio, 2021) and the impersonal nature of telehealth (Johnston, 2019), it has been shown to overcome the barriers to accessing face-to-face therapy by reducing time, cost and travel for parents (Cole et al., 2019). Prior to telehealth, home-based interventions were mostly provided through home programmes which have been identified as being potentially stressful for parents who needed to take on a therapy role at home (Longo et al., 2020).

Most literature suggests that parents desire a hybrid model of face-to-face and telehealth services. A review of evidence by Zylstra (2013) revealed that six out of nine studies showed parents to be increasingly satisfied with telehealth when offered in combination with face-to-face services. Yet, as no known qualitative studies exploring parental perspectives of telehealth for DCD have been published, little is known about the potential for telehealth for this population. Further research is needed to explore the subjective experiences of parents engaging in telehealth as they possess an unmatched understanding of their child’s needs.

## Methods

### Study context and design

This study draws on principles of phenomenology which is defined as ‘the science of describing what one perceives, senses, and knows in one’s immediate awareness and experience’ (Moustakas, 1994:26). Phenomenology raises important academic, intellectual and procedural issues while questioning the basis and standing of knowledge (Carpenter and Suto, 2008). A qualitative design was employed to enable a deep understanding of parents’ experience using telehealth, its impact on family life and the triumphs and challenges they faced. The focus of this research was to explore the parental experiences of telehealth following their engagement with a telehealth programme called ‘Octobox’ (Kennedy, 2021). This programme was created by a private paediatric occupational therapist in Ireland and is aimed at children between 5 and 9 years. The activities aim to develop a child’s fine and gross motor skills, critical thinking, listening and executive functioning in a fun and interactive way. This is demonstrated through asynchronous online videos and specifically designed crafts received by parents as part of the programme. Parents received three themed Octobox packs to complete with their child over the course of 12 weeks with approximately 1 hour of activities to complete each week.

### Participants

Ethical approval for this study was obtained from the Social Research Ethics Committee at University College Cork. Purposive sampling was used to recruit participants through the paediatric occupational therapist who developed the ‘Octobox’ programme. This person performed a gatekeeper role for recruitment for this study. Participant selection by the gatekeeper was guided by the following inclusion and exclusion criteria (see Table 1).

**Table 1.** Inclusion and exclusion criteria.

Inclusion	Exclusion
Parent(s) of a child with a diagnosis of DCD/dyspraxia	Parent(s) of a child without a diagnosis of DCD/dyspraxia
Parent(s) give consent to participate in the study	Parent(s) do not give consent to participate in the study
Parent(s) received ‘Octobox’ programme	Parent(s) did not receive ‘Octobox’ programme
Child of parent(s) is aged 5–9 years when ‘Octobox’ was completed	Child of parent(s) is not aged 5–9 years when ‘Octobox’ was completed

In this study, four children with a diagnosis of DCD also had a co-occurring diagnosis. These include Dyslexia, Autism Spectrum disorder (ASD) and Attention Deficit Hyperactivity Disorder (ADHD). To minimise the risk of bias from the gatekeeper, the following steps were undertaken. The gatekeeper contacted all parents who met the inclusion criteria by email. Interested parents’ contact details were then provided to the researchers. An information sheet was emailed to these parents highlighting the purpose of the study and a consent form was completed prior to the interview process. Participants were informed that they could withdraw from the study without reason or consequence at any given time up to 2 weeks after data collection.

### Data generation

Individual semi-structured interviews were conducted with eight parents. This approach facilitated the collection of subjective responses from participants (McIntosh and Morse, 2015) and elicited the most salient information for the researchers by allowing for rephrasing of questions (Irvine et al., 2013). The developed interview schedule aimed to capture (1) parents’ experiences of telehealth, (2) opportunities and challenges presented by telehealth and (3) parental preferences of service delivery. Additional probe questions were included within the eight set of questions to ensure further depth of response from participants. At the beginning of each interview, verbal consent was also obtained from participants. All interviews were conducted remotely through the online video conferencing software, Microsoft Teams™. Interview duration ranged from 15 to 45 minutes and was audio recorded and transcribed verbatim. Identifying information pertaining to the participants was removed during the transcription process. Pseudonyms were used for each participant and anonymised transcripts were stored on a university-recognised secure google drive platform accessible only to the researchers.

### Data analysis

Data generated were subjected to thematic analysis enabling the researchers to identify, analyse and interpret patterns of meaning (‘themes’) within the qualitative data. This approach involved three stages as detailed by Clarke and Braun (2017). In the first stage, the researchers familiarised themselves with the data during data collection through interview transcription. During the second stage, codes were generated to gain a deeper understanding of the data. Concept maps were also utilised for identification of overlaps and linkages between codes which assisted with the later stage of the analytic process (Carpenter and Suto, 2008). In the final stage, the generated codes were sorted into potential themes and defined by the researchers. These stages helped the researchers to analyse and effectively communicate the data to produce this report.

### Rigour and trustworthiness

To assure the credibility of the study, member-checking was conducted by participants during the transcription process to ensure accuracy of the data (Stahl and King, 2020). An audit trail was retained throughout to ensure study dependability (Houghton et al., 2013). Furthermore, the use of reflexive journals and iterative discussions ensured a degree of rigorous for this study (Houghton et al., 2013).

## Findings

The eight participants that were recruited and participated in this study were mothers of children with a diagnosis of DCD aged 5–9 years. Participant characteristics are shown in Table 2.

**Table 2.** Participant characteristics.

Pseudonym	Age category	Marital status	Other children	Rural/urban	Previously accessed public occupational therapy services
Sheila	35–45	Married	Yes	Urban	Yes
Deirdre	35–45	Married	Yes	Urban	Yes
Leanne	35–45	Married	Yes	Urban	Yes
Jane	35–45	Married	Yes	Urban	Yes
Nicole	35–45	Married	Yes	Urban	Yes
Ciara	35–45	Married	Yes	Urban	Yes
Jenny	40–50	Married	Yes	Rural	Yes
Eileen	35–45	Married	Yes	Rural	Yes

Three themes and relevant subthemes emerged from data analysis which provide greater insight into the world of parents of children with DCD: (1) parents in the dark, (2) telehealth in the family and (3) telehealth: what success looks like.

### Parents in the dark

The period before engagement in telehealth was described by participants as a time where they felt in the dark surrounding their child’s needs. Participants reported that these feelings were magnified by the lack of support and intervention offered by the public occupational therapy system. Telehealth opened parents’ eyes to their child’s strengths and challenges for the first time. Subthemes include chaos in the absence of services, accessing services yet lacking understanding and a matter of empowerment.

#### Chaos in the absence of services

Each participant experienced a lack of support by public occupational therapy services prior to their engagement in telehealth. These included reduced access to quality services, individualised intervention and receiving a timely diagnosis. Participants reported their concerns surrounding their child’s needs were disregarded by healthcare professionals:

. . . .we’ve gone through a couple doctors and they kind of just said he was too young kind of thing just kind of pawned us off a little bit. (Eileen)

All participants reported having experienced waiting lists and varying levels of intervention:

In our own case my son is 10. We have been on a waiting list since he was four. He has never seen an occupational therapist in the public system. (Deirdre)

In the absence of services, participants reported having resorted to ‘guessing and Googling’ (Nicole) as they were desperate to find answers and be proactive as parents. Given the barriers to service accessibility, participants reported feeling left with no choice but to use online platforms such as YouTube and Pinterest to replace the role of therapy. The lack of support associated with the absence of services left parents feeling overwhelmed comparing it to ‘doing another university course’ (Deirdre).

#### Accessing services yet lacking understanding

All participants reported a lack of quality public services fuelled their transition to private occupational therapy. Participants reported feeling forced into going down the private route if they wanted to access support for their child:

A problem with receiving services in Ireland that I have found is that parents are often waiting for professional intervention, and you have a choice of going privately or you have a choice of continuing to wait. (Deirdre)

Participants reported that by accessing these services, they were able to address their child’s needs early on and ensure continuity of therapy. The transition to accessing private services provided participants with relief and optimism. However, not all participants felt empowered when accessing support for their child through private services. Once they received intervention, participants reported minimal insight into their child’s needs as they received limited updates on their child’s progress:

. . .the way that the OT works is that you just drop them at the door and then you would get a report. . .after eight weeks or whatever. So, I kind of didn’t really know. . .(Jane)

The level of knowledge and education of participants surrounding their child’s needs was seen to be essential to feeling empowered as a parent.

#### A matter of empowerment

The majority of participants in this study reported empowerment as being innate to the telehealth process. All participants reported that their involvement in therapy was associated with an increased understanding of their child’s needs. Prior to engagement in telehealth, participants reported that a deficit-based approach was adopted towards their child’s abilities, describing how they placed them in ‘cotton wool’ (Eileen) for fear they may injure themselves. Witnessing their child engage in telehealth was described by parents as a watershed moment in understanding their child’s needs. Participants reported that after the programme they felt more inclined to give their child increased independence from zipping up their own coat to helping around the house. Participants reported that they were previously unaware of their child’s level of motor skills as these tasks were typically completed in the classroom:

. . .the things that were more expected that were quite easy like she was struggling with those as well so I could kind of see like wow she is kind of behind in those different areas that I didn’t realise before. . . (Jane)

Participants revealed varying responses to empowerment. Although all participants reported having an increased understanding of their child’s needs after telehealth, this did not always result with feeling empowered as a parent. Participants described how guidance from a healthcare professional was preferable to carry out the intervention as they felt assuming the role of therapist was out of their scope as a parent:

I don’t really feel qualified to do it in a way other than as a mother. (Deirdre)

Limited knowledge and feeling insufficient to coach their children through therapy were associated as barriers to feeling empowered as a parent.

### Telehealth in the family

The flexibility of telehealth was valued by participants as it allowed for a seamless transition into their family life. However, the arrival of telehealth for families was not without its challenges. Participants described the impact of telehealth on the child–parent relationship and the presence of siblings on their child’s engagement with the programme. Subthemes included the fabric of family life, therapy comfort and timing, blurring the roles and sibling involvement.

#### The fabric of family life

Participants reported that the flexibility of telehealth suited the demands of family life. Participants did not have to source childcare for their other children or face the additional charges for travel with telehealth. Participants valued how they could engage in telehealth wherever and whenever they wanted to best suit their family routine:

I just love when I can literally just sit at home and turn on the computer. I don’t have to drive half an hour to meet somebody. We’re waiting. . …and then it’s another half an hour to come home and I have to get somebody to mind my other small man. (Sheila)

The home was seen as superior to the clinic environment for participants. Participants reported that their child experienced increased levels of comfort when engaging in therapy in the home. Attending in-clinic therapy was described as a stressful experience for participants due to their busy schedules as their child was often attending multiple therapies.

#### Therapy comfort and timing

Participants reported that the flexibility of telehealth enabled them to implement the programme at a time that best suited their child. Parents were freed of concerns about appointments clashing with school or work. Given the set times of clinic appointments, participants reported that it was impossible to predict the child’s mood on that day. Willingness to engage was reported to be impacted by the child’s behavior which meant that clinic interventions were often ineffective:

. . .if she’s in a bad mood and she’s not going to get anything out of that therapy session. . .cause she’s just gonna refuse to do it. . . . Whereas with Octobox if she’s just having a bad day we just won’t do it that day. You know we can do it tomorrow. (Jane)

With telehealth, participants described how they were able to pick a time where the child would engage and perform well. One parent described how she could implement the programme to the timing of her child’s medication for optimal performance:

I didn’t have to worry about making sure at that time my child was on a short acting Ritalin so I didn’t have to worry about trying to like time his dose so that it wouldn’t be ending during the middle of his OT session. (Deirdre)

Participants reported that telehealth removed previous worries with in-person therapy such as mealtimes and bathroom breaks as they were in the comforts of their own home.

#### Blurring the roles

Many participants felt implementing the telehealth programme meant they took on the role of therapist. Participants described how they disliked assuming this role as it changed the dynamic of their child–parent relationship:

I actually hate being the primary teacher of my child because it changes my relationship with my child. I’m not just his mom anymore. I have assumed this role of a person who makes you do these things to help you better and grow. (Nicole)

Parents reported how they found it difficult to transition from this role when intervention ceased. Participants described how they missed out on playing opportunities with their child as they felt they needed to prioritise therapy. Participants’ personal time in the evenings was spent researching information and therapy activities as they wanted to source the best information for their child. Whilst participants assumed this role they did not always feel trained for it and felt they were not doing an adequate job:

I always knew that I wasn’t an expert, and I could be getting something wrong, and missing an opportunity missing an important sign that something else would be more effective. (Deirdre)

Participants reported that the position of ‘theraparent’ or ‘momager’ (*Nicole*), however, was not unique to the motor skills programme as it applied to home therapy in general. The impact of telehealth on the child–parent relationship was seen to be dependent on the family dynamic and prior experiences with services. One participant discussed how they had a ‘partnership’ (Sheila) with their child given the extensive number of therapies their child is receiving. The participant reported that they had no choice but to make it work; however, they believed that parents 4 years behind them in services would have differing attitudes.

#### Sibling involvement

Participants reported mixed views on sibling involvement in telehealth. For some, involving siblings was a positive facilitator to the child’s engagement as they motivated and encouraged the child. Participants reported that the presence of siblings made the programme a fun and enjoyable experience and how older siblings could act as supervision in lieu of the parent. Furthermore, the participants reported sibling involvement increased their awareness of their child’s ability:

When he was doing it alongside his younger brother. . . .I could see that something was easy for his brother who is 3 years younger and quite difficult for him. (Leanne)

Conversely, other participants reported sibling involvement as a limitation of telehealth in the home. Sibling involvement was reported as a distraction as they viewed the programme as merely a game they wanted to join. Participants reported how they struggled to find one-on-one time with their child as they felt conscious of singling out their child from their other siblings. Participants reported how the siblings would often feel left out when told the programme was solely for their child with DCD.

### Telehealth: What success looks like

All participants highlighted the inherent value of telehealth in supporting their children with DCD. Specific to the programme in this study, participants discussed what they felt characterised an effective telehealth programme and the place for telehealth in the future. What telehealth is missing, not all telehealth is the same and a future of blended therapy were the subthemes emerging from this theme.

#### What is telehealth missing?

Participants highlighted that there were specific elements of in-person therapy they felt were missing from telehealth. The absence of healthcare professionals was described by all participants as the main limitation of engaging in a telehealth service. Participants reported not having a therapist physically present was difficult as they lacked confidence in their abilities to implement the programme and felt they may misinterpret information:

It is very much often the health care practitioners are taken out of the equation kind of and the parent and the child are left basically on their own. (Shelia)

The role of the therapeutic environment was discussed by participants and their experiences of home versus clinic therapy. While all participants reported the unique value of home therapy, many participants described how the abundance of resources, equipment and space in the clinic were absent from telehealth. Participants also reported how communicating virtually impacted the therapeutic relationship and thus missed the value of face-to-face interaction with the therapist.

#### Not all telehealth is the same

Specific components of ‘Octobox’ were reported by participants as being particularly effective for them as parents. All participants reported that the video component of the programme was its unique selling point. Participants reported how the videos allowed for flexibility of use with the pause and rewind feature. For many of the children, particularly those with a dual diagnosis of ASD, participants reported the visual element combined with the programme worked extremely well. Participants valued the fun and enthusiastic nature of the therapist in the video demonstrations. Participants also reported having the just right challenge and a reward element were essential for motivating their child, avoiding frustration and providing their child with a sense of achievement:

There was kind of a reward at the end of it when he completed it that he had actually made something himself. . . . that was really nice to see that kind of achievement in itself and that. . . . . .Pride, whenever he had actually managed to complete a task. (Eileen)

Feeling supported as a parent was seen as essential by the participants when engaging in telehealth. Participants reported they valued how the programme was created by an occupational therapist as they felt this increased the programme’s validity. The ease of programme setup meant that many participants did not reach out; however, they reported they felt they could access advice or support from a therapist if needed.

#### A future of blended therapy

All participants felt telehealth did not take the place of occupational therapists and would be most beneficial when used in conjunction with face-to-face therapy:

I don’t want to see telehealth as something that is brought into the public domain without people having the practitioners in the background. (Sheila)

Telehealth as a scalable solution to the barriers experienced by participants when accessing services emerged from the interviews. Participants felt that telehealth could provide parents with the opportunity to be proactive while being on a waitlist as it could provide them with insight into their child’s needs. Participants reported this programme would have been invaluable when they were unable to access services for their child. However, many participants felt that an initial face-to-face appointment prior to receiving telehealth services to understand their child’s needs and build therapeutic rapport would facilitate parent engagement in the programme. Participants also reported that the telehealth programme could be used to ‘stretch out time between visits’ (Ciara) while reinforcing the work the child has done in therapy at home.

## Discussion and implications

This study provides a greater insight into the experiences faced by parents of children with DCD in Ireland. All participants reported a lack of support by public occupational therapy services prior to their engagement in telehealth. One participant reported waiting 6 years and counting for their child to receive occupational therapy intervention. This comes as no surprise as due to rising costs publicly funded services grapple to address the increasing number of children presenting with motor delays (Hurtubise et al., 2021) and prioritise those with more severe motor difficulties (Miller et al., 2008). This finding is significant as a lack of early intervention has the potential to negatively impact children’s development (Camden and Silva, 2021) and increase stress levels in these families (Miller et al., 2008). It is clear that the public healthcare system needs to adopt a service model that delivers intervention not solely face-to-face to combat rising costs and increasing waitlists.

The transition to telehealth revealed how parents felt increasingly empowered. Participants indicated that telehealth allowed them to be an active participant in their child’s therapy, whereas previous models of service delivery (e.g. clinic therapy, school) saw the therapist take the lead. Participants described how telehealth encouraged them to problem solve solutions with their child as opposed to previously completing tasks for them when difficulties arose. A study by Wallisch et al. (2019) found that parents experienced increased confidence to try new strategies and problem-solving situations following telehealth intervention. For participants, telehealth enhanced compatibility with family life. Participants matched the timing of therapy with the child’s window of tolerance and mood in contrast to a clinic setting where appointments were difficult to reschedule. As reported in previous studies (Cole et al., 2019; Wallisch et al., 2019), telehealth was seen as a service delivery model that met the needs of both the child and the family. For participants, telehealth removed the barriers with face-to-face appointments including sourcing a babysitter, time, travel and cost. In line with previous studies, technology increased the accessibility and cost-effectiveness of services (Little et al., 2018). For many participants, telehealth was the only means of receiving intervention for their child given the barriers to service accessibility in the public system. Practitioners therefore must consider the potential for telehealth to be used for children with DCD as despite intervention requirements (Blank et al., 2019), they are subject to a shortage of services (Soriano et al., 2015). Telehealth may be beneficial to increase the duration between face-to-face intervention by allowing for reinforcement of therapy activities in the home. However, while empowerment for many participants was associated with engagement in telehealth, for others this involved assuming the role of therapist and blurring their role as a mother. This is significant for practitioners as unclear expectations about roles have been associated with increased parental stress (Coyne, 2015). This shift in family dynamics suggests the need to refocus occupational therapy intervention to place a greater emphasis on parental education and empowerment.

The findings of this study and previous research suggest that parents would prefer a blended approach to therapy (combination of telehealth and face-to-face) integrated into one service delivery model in the future (Johnston, 2019). Participants appreciated the unique value of in-person therapy including support, resources, equipment and space and felt an initial clinic assessment prior to commencement with telehealth services would be beneficial. Karlsudd (2008) suggests that such an approach may increase parental engagement with remote services by establishing therapeutic rapport. To inform the development of a blended approach to therapy, practitioners need to be equipped with the digital fluency, knowledge and technical skills to meet parents’ and children’s needs in using remote technology platforms. Therapists should therefore reflect on what components of practice would best suit a remote platform and how the therapeutic use of self can be translated into remote service delivery. However, this change needs to happen on a systemic level as the outdated public system is currently not agile enough to the changing needs of parents of children with additional needs. As novel programmes like ‘Octobox’ are currently only being offered by private practitioners, services run by statutory providers may risk becoming obsolete as they offer less flexibility and choice in the therapeutic process. Further in-depth research is therefore needed to examine the growing privatisation of allied healthcare and the role of telehealth to combat such issues in today’s increasingly virtual world.

### Limitations

This programme focused on asynchronous videos; therefore; further research examining alternative telehealth methods is imperative to illuminate parents’ overall experiences of remote service delivery for children with DCD. The small sample size recruited certainly limits the generalisability of our research findings as does the specificity of the telehealth solution at the heart of this study. Furthermore, information surrounding the socio-economic circumstances of parents were not collected. Further research to examine socio-economic factors and the impact of severity of disabilities for parents of children with DCD across various occupational domains is therefore warranted. This study also included children with dual diagnoses; therefore, further studies examining children with a sole diagnosis of DCD may be beneficial. However, it is important to note that DCD is commonly associated with other neurodevelopmental disorders (Lino and Chieffo, 2022). Despite these limitations, the findings suggest that further studies with more diverse services, addressing different needs and using other technologies, are needed.

## Conclusion

The barriers associated with service accessibility for children in Ireland have led many occupational therapists to re-evaluate the role of technology in their practice. The results of this study provide preliminary support for the use of telehealth to extend services to children with DCD and their families by enabling parents to access therapy more conveniently in terms of cost, time and travel. The absence of healthcare professionals in-person however was felt by families and all participants agreed that telehealth should complement rather than replace face-to-face therapy with therapists. Nonetheless, the potential for telehealth to positively contribute to the lives of children with DCD and their families in the future is clear.

**Key findings**

- Telehealth is more convenient for families in terms of cost, time and travel.
- A blended approach to therapy is favoured by parents.
- Parental empowerment requires building parents cumulative skills and experiences.

**What the study has added**

Qualitative research findings providing a greater insight into the experiences faced by parents of children with DCD in Ireland and the potential benefits of telehealth to overcome barriers to service accessibility.
